# Alterations in the Spectrum of Spontaneous Rifampicin-Resistance Mutations in the *Bacillus subtilis rpoB* Gene after Cultivation in the Human Spaceflight Environment

**DOI:** 10.3389/fmicb.2018.00192

**Published:** 2018-02-14

**Authors:** Patricia Fajardo-Cavazos, Joshua D. Leehan, Wayne L. Nicholson

**Affiliations:** Department of Microbiology and Cell Science, University of Florida, Gainesville, FL, United States

**Keywords:** antibiotic resistance, mutation, *rpoB*, rifampicin, *Bacillus subtilis*, spaceflight

## Abstract

The effect of *Bacillus subtilis* exposure to the human spaceflight environment on growth, mutagenic frequency, and spectrum of mutations to rifampicin resistance (Rif^R^) was investigated. *B. subtilis* cells were cultivated in Biological Research in Canister-Petri Dish Fixation Units (BRIC-PDFUs) on two separate missions to the International Space Station (ISS), dubbed BRIC-18 and BRIC-21, with matching asynchronous ground controls. No statistically significant difference in either growth or in the frequency of mutation to Rif^R^ was found in either experiment. However, nucleotide sequencing of the Rif^R^ regions of the *rpoB* gene from Rif^R^ mutants revealed dramatic differences in the spectrum of mutations between flight (FL) and ground control (GC) samples, including two newly discovered *rpoB* alleles in the FL samples (Q137R and L489S). The results strengthen the idea that exposure to the human spaceflight environment causes unique stresses on bacteria, leading to alterations in their mutagenic potential.

## Introduction

In contrast to the classical view that mutations are random, a growing body of evidence indicates that exposure to environmental stresses in microbes can alter both the mutation rate and the mutagenic spectrum, supplying an increased variety of mutational outputs for selection to operate on and hence shaping the evolutionary trajectory of organisms (Nicholson and Maughan, 2002; Foster, 2007; Nicholson and Park, 2015; Maharjan and Ferenci, 2017b). This phenomenon, dubbed Stress-Induced Mutagenesis (SIM), has been most eloquently demonstrated in the recent work of Maharjan and Ferenci (b). *E. coli* cells were grown in chemostats under 5 different stresses, (limitation for carbon, phosphate, nitrogen, oxygen, or iron) or in a nutrient-rich environment, and loss-of-function mutations in the *cycA* gene leading to cycloserine resistance (Cyc^R^) were quantified, isolated, and subjected to fitness measurements (Maharjan and Ferenci, 2017b). Although only 2 out of 5 stresses led to a significant increase in the mutation rate to Cyc^R^, all stresses resulted in distinct spectra of mutations in *cycA* conferring a variety of alterations in fitness of the resulting mutants (Maharjan and Ferenci, 2017b).

The human spaceflight environment presents its own unique set of physical stressors, including cosmic radiation, microgravity, vibration, electromagnetic fields, and altered atmospheric compositions. Numerous studies have explored how microorganisms respond physiologically to spaceflight exposure (reviewed extensively in Rosenzweig et al., 2014; Taylor, 2015). However, very few studies have asked whether microbial exposure to spaceflight might lead to SIM (Yatagai et al., 2000; Fajardo-Cavazos and Nicholson, 2016). Such studies are relevant to astronaut health risk assessment, given the role that microbial SIM may play in the emergence of antibiotic resistance or modifications to the astronaut microbiome. Long-duration human missions through interplanetary space to the Moon, near-Earth asteroids, or Mars are currently being planned (NASA, 2015), and both the National Research Council (NRC) and the International Space Exploration Coordination Group (ISECG) have assigned a high priority to studies aimed at better understanding astronaut health risks during space exploration. NASA has implemented a three-phase approach to its long-term goal of the human exploration of Mars^1^. The first phase, to be accomplished in the present-to-mid-2020’s time frame, is dubbed the “Earth Reliant” phase, focusing on research aboard the International Space Station (ISS), currently the only microgravity platform for the long-term testing of crew health systems and technologies needed to decrease reliance on Earth. To date, only two studies have been performed exploring the notion that exposure of microbes to the stresses of the human spaceflight environment might result in SIM. First, *B. subtilis* spores containing a plasmid encoding the *Escherichia coli rpsL* gene were exposed to spaceflight in space station *Mir* vs. matched ground controls (Yatagai et al., 2000). The frequency of mutations in *rpsL* leading to spectinomycin resistance (Spc^R^) was found not to be significantly different between spaceflight and ground control samples, but sequencing of the mutant *rpsL* genes from 25 spaceflight and 38 ground control samples showed distinct differences in the spectrum of mutations leading to Spc^R^ (Yatagai et al., 2000). Second, the frequency and spectrum of chromosomal mutations to rifampicin resistance (Rif^R^) was measured in *Staphylococcus epidermidis* cells flown aboard the ISS and compared to matched ground controls (Fajardo-Cavazos and Nicholson, 2016). In this experiment the frequency of mutation to Rif^R^ was observed to be significantly (24-fold) greater in the spaceflight samples. Sequencing of the *rpoB* gene from 67 spaceflight and 57 ground control Rif^R^ mutants also showed that the spectrum of mutations to Rif^R^ was clearly different in the flight vs. the ground control samples (Fajardo-Cavazos and Nicholson, 2016).

From our previous results (Fajardo-Cavazos and Nicholson, 2016), we concluded that Rif^R^ was a good model to investigate SIM in spaceflight for a broad variety of microorganisms. Resistance to rifampicin derives from mutations within a small region of the *rpoB* gene encoding the β-subunit of RNA polymerase (Wehrli et al., 1968; Jin and Gross, 1988). This region corresponds to the Rif binding site of β (Campbell et al., 2001) and is highly conserved among prokaryotes (Severinov et al., 1993). Single-nucleotide substitutions in *rpoB* leading to Rif^R^ have been shown to alter the global patterns of transcription, and resulting phenotypes, in several bacteria including *Neisseria meningitidis, Streptomyces lividans, Bacillus subtilis, Mycobacterium tuberculosis*, and *Staphylococcus aureus* (Hu et al., 2002; Maughan et al., 2004; Perkins and Nicholson, 2008; de Knegt et al., 2013; Colicchio et al., 2015; Villanueva et al., 2016). It is thus possible that mutation to Rif^R^ could alter the response of microbes to the spaceflight environment.

For the present study we chose to use as the test organism the Gram-positive bacterium *B. subtilis*, which possesses several advantages as a model organism: (i) it is easily cultivated and amenable to genetic manipulation; (ii) it forms dormant spores, making it an easy system to prepare for spaceflight; (iii) it is the best-studied Gram-positive bacterium; and (iv) development of its genetics, genomics, and molecular biology is highly advanced. Thus, using the well-developed *B. subtilis* system will facilitate the investigation in further detail of any potential novel mutations isolated in *rpoB*.

## Materials and Methods

### Bacterial Strain, Media, and Growth Conditions

The strain used was *Bacillus subtilis* strain 168 (*trpC2*) from the authors’ laboratory collection. Medium used throughout was trypticase soy yeast extract (TSY) medium consisting of (g/L): tryptone, 15; soytone, 5; NaCl, 5; yeast extract, 3; K_2_HPO_4_, 2.5; glucose, 2.5; final pH 7. For semisolid plates, agar was added to TSY at 15.0 g/L. For FL and GC experiments, glycerol was added to TSY liquid medium to 10% (v/v) final concentration, resulting in TSYG medium. As appropriate, the antibiotic rifampicin (Rif; Sigma–Aldrich) was added to TSY at a final concentration of 5 μg/mL. *B. subtilis* spores were routinely prepared by cultivation in liquid Schaeffer Sporulation Medium (Schaeffer et al., 1965) at 37°C with vigorous aeration. The culture was harvested when phase-contrast microscopic examination revealed that it consisted of >90% free spores, usually after 3–4 days of incubation.

### Sample Preparation

Spores were purified by lysozyme treatment, buffer washing, and heat shock (80°C, 10 min) as described previously (Nicholson and Setlow, 1990). Spores were determined by phase-contrast microscopy to be >99% free of cell debris and unsporulated cells, and were stored at 4°C in deionized water. The working suspension of spores contained 10^8^ colony-forming units (CFU) per mL. Aliquots of 0.1 mL (∼10^7^ CFU) of the suspension were applied to the bottoms of sterile 60-mm diameter Petri dishes (Falcon Cat. No. 1007) and air-dried for 48–72 h at room temperature prior to use.

### BRIC Spaceflight Hardware

The experiments described here utilized Biological Research In Canisters (BRIC) spaceflight hardware, which has been described in detail previously (Paul et al., 2012). BRIC canisters can accomodate up to six samples. In these experiments, each BRIC was loaded with 5 Petri Dish Fixation Units (PDFUs). Each PDFU was loaded with a 60-mm Petri dish containing air-dried spores, and sterile TSYG medium was loaded into a separate reservoir. To prevent contamination, all reagents and equipment used were sterilized prior to use and PDFUs were assembled using aseptic technique within a Class 2 Biological Safety Hood. In the sixth sample chamber of each BRIC was placed a HOBO R temperature data logger (Onset Computer Co., Bourne, MA, United States). Post-flight asynchronous GC experiments were conducted using the same hardware and configuration as in the flight experiment.

### Flight (FL) and Ground Control (GC) Timelines

*Bacillus subtilis* samples were flown on two separate experiments to the ISS, the 18th and 21st BRIC missions to space, designated BRIC-18 and BRIC-21. FL samples were launched on the 3rd and 6th SpaceX Cargo Resupply missions to the ISS (SpaceX-3 and SpaceX-6 CRS, respectively), using the Falcon 9 rocket and Dragon capsule configuration. Pertinent information regarding the activity timelines of these two missions can be found in **Table 1**. Growth of samples was initiated in-flight and samples incubated for 122 h (BRIC-18) or 25 h (BRIC-21) before transfer to the onboard -80°C freezer. Frozen samples were returned to Earth in the Dragon capsule and were maintained in the frozen state until return to KSC for de-integration and further processing.

**Table 1:** Schedule of activities for BRIC-18 and BRIC-21 Flight (FL) and Ground Control (GC) experiments^∗^.

Actual or simulated activity	BRIC-18				BRIC-21			
	FL		GC		FL		GC	
	Day	Date^∗^	Day	Date	Day	Date	Day	Date
Integration/handover	–7	140411	–7	140613	–3	150411	–3	150616
Launch	0	140418	0	140620	0	150414	0	150619
Docking at ISS	3	140421	2	140622	3	150417	3	150622
Unpacking/stowage	4	140422	4	140622	4	150418	4	150623
Injection of growth medium	12	140430	12	140702	6	150420	6	150625
Transfer to -80°C	17	140505	17	140708	7	150421	7	150626
Undocking/splashdown	30	140518	30	140720	37	150521	37	150726
Arrival at KSC	35	140523	35	140725	42	150528	42	150802
Deintegration	46	140603	46	140805	46	150601	46	150806

*^∗^All dates are in the format year-month-day.*

Asynchronous GC experiments were performed in BRIC canisters according to the timelines determined during the FL experiments (**Table 1**). Samples were incubated in the KSC ISS Environmental Simulation Chamber following the temperature regimes recorded during the flights (Fajardo-Cavazos and Nicholson, 2016; Morrison et al., 2017) and growth was terminated by transfer to a -80°C freezer, where samples were stored until further processing.

### Post-experiment Sample Processing

In the BRIC-18 experiment, both FL and GC BRIC canisters were transferred from storage at -80°C to a +4°C refrigerator and samples allowed to thaw completely overnight. Liquid samples in Petri dishes were recovered from the PDFUs, transported to the laboratory on ice and further processed immediately. In the BRIC-21 experiment, both FL and GC samples were recovered from BRIC canisters in the frozen state, transported on dry ice to the laboratory, and stored at -80°C for later processing. Frozen samples were then thawed on ice, resuspended and processed immediately. In all instances, cells were detached from interior plate surfaces and resuspended using sterile disposable rubber spatulas. Resuspended cultures were transferred to sterile 15-mL conical centrifuge tubes, and the total recovered volume was measured. For viable counts, aliquots from cultures were diluted serially tenfold in TSY medium, dilutions plated on TSY plates, and colonies counted after incubation at 37°C for 24 h to obtain CFU/mL. To obtain the total CFU per PDFU, the CFU/mL were multiplied by the volume of liquid recovered. To select for Rif^R^ mutants, the remainder of each culture was concentrated by centrifugation, plated without dilution onto TSY + Rif plates, and colonies counted after incubation at 37°C for 24–48 h. The frequency of mutation to Rif^R^ was calculated by dividing the total number of Rif^R^ mutants by the total number of viable cells from each sample. Individual Rif^R^ mutants were streak-purified on TSY + Rif plates and stored at -70°C in TSY + 25% glycerol, then processed for DNA sequencing.

### DNA Sequencing and Analyses

Primers used for PCR amplification of two Rif^R^ regions of the *B. subtilis rpoB* gene are listed in **Table 2**. The corresponding *rpoB* regions were amplified directly from cells using 3 μL of culture from their respective glycerol stocks as DNA template, using the GoTaq^®^ PCR kit (Promega, Madison, WI, United States) and the following thermal cycling conditions: 95°C, 2 min for the initial denaturation step, then 36 cycles of (95°C, 30 s; 55°C, 1 min; 72°C, 2 min), followed by a final elongation step (72°C, 5 min). PCR amplicons were purified and sequenced at the University of Florida Interdisciplinary Center for Biotechnology Research (BRIC-18) or at GeneWiz LLC (South Plainfield, NJ, United States) (BRIC-21). Multiple *rpoB* sequences were aligned using the online Clustal Omega server^2^ (Li et al., 2015) to identify the position of mutations relative to the wild-type *rpoB* sequence obtained from *B. subtilis* laboratory strain 168. In order to confirm the identity of novel (i.e., never-before-isolated) *rpoB* mutations, the corresponding region of the newly sequenced *rpoB* allele was amplified by PCR from each mutant and the resulting amplicon was introduced by transformation into competent cells of Rif^S^ *B. subtilis* strain WN547 using standard procedures as described previously (Boylan et al., 1972; Nicholson and Maughan, 2002). From selected Rif^R^ transformants, the corresponding region of *rpoB* was again PCR amplified and sequenced to confirm that the mutation had been transferred.

**Table 2:** Oligonucleotide primers used for amplification of *B. subtilis rpoB* regions.

Primer	Sequence 5 → 3′	*rpoB* region amplified
Bsu rpoB-24F	CGCATGATTTGAGGGG	N-cluster
Bsu rpoB+737R	GGCGGCTCTCCAGG	N-cluster
Bsu rpoB+1319F	CGAATACAATACGCCTCAGC	Clusters I, II, III
Bsu rpoB+2000R	CCTGATACGTATTCCATACC	Clusters I, II, III

### Statistical Analyses

Non-parametric statistical parameters and tests of significance (Kruskal–Wallis) were computed on log_10_-transformed data using Kaleidagraph version 4.5.2 (Synergy Software, Reading, PA, United States).

## Results

### Temperature Data in FL vs. GC Experiments

Temperature data were logged at 10-min intervals during the FL and GC experiments and a summary of the data is presented in **Table 3**. A detailed presentation of the temperature data can be found elsewhere (Fajardo-Cavazos and Nicholson, 2016; Morrison et al., 2017).

**Table 3:** Temperatures recorded during BRIC-18 and BRIC-21 FL and GC experiments.

Mission	FL	GC	Reference
BRIC-18	25.1 + 0.12°C	24.8 + 0.16°C	Fajardo-Cavazos and Nicholson, 2016
BRIC-21	22.8 + 0.07°C	22.8 + 0.21°C	Morrison et al., 2017

### Growth in FL vs. GC Samples

**FIGURE 1:**
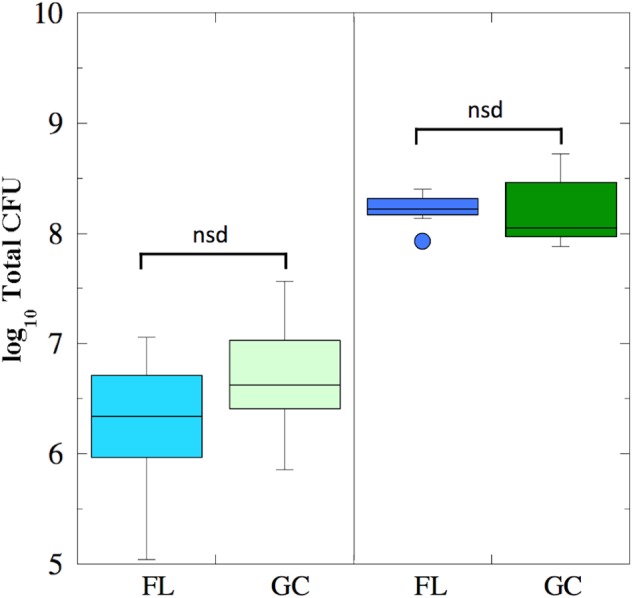
Box plots depicting growth (presented as log_10_ Total CFU) of *B. subtilis* FL (cyan and blue boxes) and GC (light and dark green boxes) samples during the BRIC-18 (left panel; *n* = 5) and BRIC-21 (right panel; *n* = 7) experiments. nsd, not significantly different (Kruskal–Wallis, *P* > 0.05).

Viable cell counts were determined for both FL and GC samples recovered from the BRIC-18 and BRIC-21 experiments (**Figure 1**). Because the data was determined not to be normally distributed, it was analyzed using non-parametric statistics. In the BRIC-18 experiment, cells grew to average total CFU per PDFU of 3.98 × 10^6^ (FL) and 1.10 × 10^7^ (GC); these values were determined not to be significantly different (**Figure 1**). Ground-based growth kinetic experiments revealed that the rather low cell yield in the BRIC-18 mission was due to the prolonged incubation time (122 h) (data not shown). For the BRIC-21 mission, an increased cell yield was needed to provide sufficient sample mass for additional antibiotic susceptibility testing (Morrison et al., 2017) and RNA-seq analysis (to be published elsewhere). In ground experiments preparatory for the BRIC-21 mission, it was determined that an incubation time of 25 h resulted in higher cell yields, and that 25-h cultures were in the late-logarithmic to early stationary phase (data not shown). Accordingly, statistical comparison of the total CFU obtained between the BRIC-18 and BRIC-21 flights uncovered that cells grew to significantly higher titers in the BRIC-21 experiment, to an average total CFU per PDFU of 1.72 × 10^8^ (FL) and 2.13 × 10^8^ (GC) (*P* < 0.005). As found in the BRIC-18 mission, FL vs. GC titers within the BRIC-21 mission were determined to be not significantly different (**Figure 1**).

### Mutation Frequencies to Rif^R^ in FL vs. GC Samples

**FIGURE 2:**
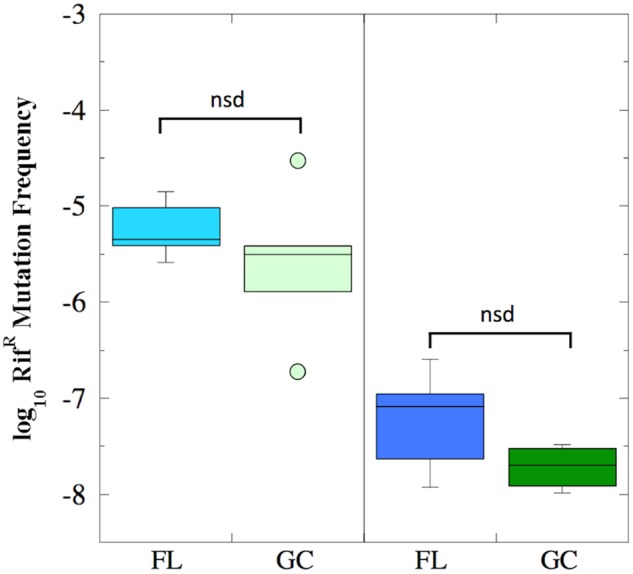
Box plots depicting the frequency of Rif^R^ mutations (presented as log_10_ values) of *B. subtilis* FL (cyan and blue boxes) and GC (light and dark green boxes) samples during the BRIC-18 (left panel; *n* = 5) and BRIC-21 (right panel; *n* = 6) experiments. nsd, not significantly different (Kruskal–Wallis, *P* > 0.05).

The mutation frequency to Rif^R^ was determined for both FL and GC samples recovered from the BRIC-18 and BRIC-21 experiments (**Figure 2**). Because the data were determined not to be normally distributed, they were analyzed using non-parametric statistics. In the BRIC-18 experiment, cells exhibited an average mutation frequency of 6.9 × 10^-6^ (FL) and 7.54 × 10^-6^ (GC); these values were determined to be not significantly different (**Figure 2**). In the BRIC-21 experiment, cells exhibited an average mutation frequency of 6.11 × 10^-8^ (FL) and 1.94 × 10^-8^ (GC); again, these values were determined to be not significantly different (**Figure 2**). However, statistical comparison of the mutation frequencies to Rif^R^ obtained between the BRIC-18 and BRIC-21 flights revealed that cells demonstrated a significantly higher frequency of mutation to Rif^R^ in the BRIC-18 experiment (*P* < 0.007). Again, this may result from differences in the incubation times to which the cells were subjected–122 h in BRIC-18 vs. 25 h in BRIC-21–due to the well-documented phenomenon of stationary phase mutagenesis in *B. subtilis* (Robleto et al., 2007).

### Spectrum of Rif^R^ *rpoB* Mutations in FL vs. GC Experiments

**FIGURE 3:**
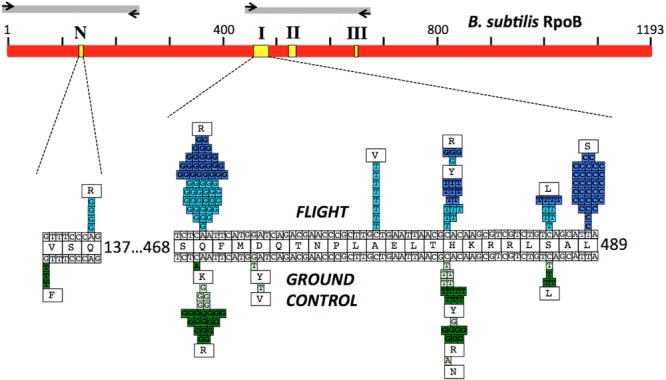
**(Top)** Schematic of the *B. subtilis* RpoB protein sequence (red bar). Yellow boxes denote the positions of the N-cluster and Clusters I, II, and III where Rif^R^ mutations have been located (Severinov et al., 1993). Gray bars denote the regions amplified by PCR and sequenced using the corresponding primers (black arrows; see **Table 2** for details). **(Bottom)** Expanded graphic summary of the distribution of Rif^R^ mutations. Shown are the N-cluster and Cluster I of the *B. subtilis rpoB* gene. The center row depicts the RpoB amino acid sequence, flanked above and below by the wild-type *rpoB* nucleotide sequence. Mutations identified in FL and GC samples are denoted above and below the central line, respectively. Each box represents an independently sequenced mutation, and the corresponding amino acid alteration is indicated. Data from both the BRIC-18 (cyan and light green boxes) and BRIC-21 (blue and dark green boxes) are presented.

The N-cluster and Clusters I, II, and III of the *rpoB* gene were PCR amplified from *B. subtilis* strain 168 and from the Rif^R^ mutants obtained from the BRIC-18 and BRIC-21 FL and GC experiments. Nucleotide sequences were determined from a total of 56 FL and 15 GC samples from BRIC-18 (**Table 4**) and from a total of 72 FL and 38 GC samples from BRIC-21 (**Table 5**). Both sets of data are summarized graphically in **Figure 3**. Examination of the data revealed several striking differences in the mutational spectrum within *rpoB* between the FL and GC samples.

**Table 4:** Summary of *rpoB* mutations leading to Rif^R^ in *B. subtilis* flight (FL) vs. ground control (GC) samples, BRIC-18 mission.

PDFU	1		2		3		4		5		Total	
Mutation	FL	GC	FL	GC	FL	GC	FL	GC	FL	GC	FL	GC
Q137R			2		2						4	0
Q469R	2		2	4	1	1	5		16		26	5
D472V										1	0	1
D472Y								1			0	1
A478V			8								8	0
H482N										1	0	1
H482R	1							1			1	1
H482Y	1				5					5	6	5
S487L	1	1					4				5	1
No change			2		4						6	0
Total	5	1	14	4	12	1	9	2	16	7	56	15

**Table 5:** Summary of *rpoB* mutations leading to Rif^R^ in *B. subtilis* flight (FL) vs. ground control (GC) samples, BRIC-21 mission.

PDFU	1		2		3		4		5		6		Total	
Mutation	FL	GC	FL	GC	FL	GC	FL	GC	FL	GC	FL	GC	FL	GC
V135F										3			0	3
Q469K												1	0	1
Q469R		2	1		4	12	5	2	11		4	1	25	17
H482R	1		1							5	1	2	3	7
H482Y	1	1		2				2			7	2	8	7
S487L			2	1		1	2	1					4	3
L489S					7		25						32	0
Total	2	3	4	3	11	13	32	5	11	8	12	6	72	38

#### N-Cluster

Historically, the only mutation conferring Rif^R^ previously identified in the N-cluster of *B. subtilis rpoB* was a G-to-T transversion causing the amino acid change V135F; this mutation was previously found at approximately equal frequencies in spores exposed either to Earth or simulated Mars conditions (Perkins et al., 2008). In the present experiments, the V135F mutation was observed only in BRIC-21 GC samples (**Figure 3** and **Table 5**). However, a novel mutation, Q137R, was detected in the N-cluster in 4 out of 56 BRIC-18 FL samples, but in none of the GC samples (**Figure 3** and **Table 4**). This novel mutation was confirmed by retransformation and resequencing as described in Materials and Methods. The Q137R mutation was found in two separate FL PDFUs, suggesting that the spaceflight environment may be conducive to its formation (**Tables 4, 6**).

**Table 6:** Distribution of classes of Rif^R^ *rpoB* mutations appearing at least once in FL and GC samples^∗^.

	FL						GC						No. of PDFUs	
PDFU number:	1	2	3	4	5	6	1	2	3	4	5	6	FL	GC
Amino acid change														
V135F											X		0	1
Q137R		X	X										2	0
Q469K												X	0	1
Q469R	X	X	X	X	X	X	X	X	X	X		X	6	5
D472V											X		0	1
D472Y										X			0	1
A478V		X											1	0
H482N											X		0	1
H482R	X	X				X				X	X	X	3	3
H482Y	X		X				X	X		X	X		2	4
S487L	X	X		X			X	X	X	X			3	4
L489S			X	X									2	0
No change found		X	X										2	0

*^∗^Data are combined from BRIC-18 and BRIC-21 missions. Each class of mutation was scored as either present (X) or absent (blank) in the corresponding PDFU. Totals are tabulated in the rightmost two columns.*

#### Cluster I

The majority of Rif^R^ mutations in both FL and GC samples were found to occur in Cluster I, in agreement with numerous prior studies. Historically, the most common amino acid changes in Cluster I leading to Rif^R^ occur at amino acids Q469, H482, and S487 (Severinov et al., 1993; Campbell et al., 2001; Nicholson and Maughan, 2002; Perkins et al., 2008). In our experiments, the spectrum and frequency of mutations appeared to be similar at these three amino acids (**Figure 3**), with the exception that a single never before isolated mutation (H482N) was identified among the GC samples from BRIC-18 (**Figure 3** and **Table 4**). However, in FL samples from the BRIC-21 experiment we found a large number of mutations (32/72) at L489, consisting exclusively of T-to-C transitions resulting in the amino acid change L489S (**Figure 3** and **Table 5**). This novel mutation was confirmed by retransformation and resequencing as described in Section “Materials and Methods.” The L489S mutation was found in two separate FL PDFUs, also suggesting that the spaceflight environment may be conducive to its formation (**Tables 5, 6**). In addition to amino acids Q469, H482, S487, and L489 we found within Cluster I two differences in the spectrum of mutations in *rpoB.* First, at amino acid D472, we observed no mutations in FL samples, but in BRIC-18 GC samples we observed two different mutations; a G-to-T transversion at the first position and an A-to-T transversion at the second position of codon D472, leading to the deduced amino acid changes D472Y and D472V, respectively (**Figure 3**). Second, no mutations were found in GC samples at amino acid A478, but in the BRIC-18 FL samples 8/59 of the total mutations consisted of a C-to-T transition at the second codon position resulting an A478V substitution (**Figure 3** and **Table 4**).

#### Rif^R^ Mutations Not Mapping to Known *rpoB* Regions

Historically, 4 regions in *rpoB* have been associated with Rif^R^ in the model organism *E. coli*, designated as the N-cluster and Clusters I, II, and III (**Figure 3**) (reviewed in Severinov et al., 1993, 1994). These clusters have been defined by the locations of Rif^R^ mutations as codons 127-135 (N-cluster), 463-489 (Cluster I), and 519-532 (Cluster II) (*B. subtilis* coordinates). To date, in *B. subtilis* Rif^R^ mutations have never been identified in Cluster III or in intervening regions. However in 2 PDFUs from the BRIC-18 mission were isolated Rif^R^ mutants for which no mutation in *rpoB* was found by sequencing the classic N-cluster or Clusters I, II, and III (**Table 4**). This phenomenon has been reported before (Ahmad et al., 2012) and raises 2 formal possibilities. First, these mutations conferring Rif^R^ might reside in *rpoB* but outside the Clusters N, I, II, or III (it should be noted that our primer sets covered only ∼38% of the complete *rpoB* sequence; **Figure 3**). Second, the mutations may be located outside *rpoB* entirely; low-level Rif^R^ has been previously reported in mutants with altered permeability or efflux (Goldstein, 2014). In any event, because these mutants appeared in FL PDFUs, they may represent new allele(s) and warrant further investigation.

### Distribution of Rif^R^ *rpoB* Mutations by PDFU

Examination of **Tables 4, 5** indicated that in some PDFUs an unusually high number of repeats of the same Rif^R^ mutant were found. For example, 16 out of 16 Rif^R^ mutants sequenced from BRIC-18 FL PDFU-5 (**Table 4**), and 12 out of 13 mutants sequenced from BRIC-21 GC PDFU-3 (**Table 5**), were Q469R. These data suggested that some of the populations in both FL and GC samples contained “jackpots,” i.e., cultures in which the progeny of early arising Rif^R^ mutants became over-represented in the final culture (Foster, 2006). We therefore analyzed the data by counting each type of mutation as either “present” (at least once) or “absent” in each PDFU, the results of which are presented in **Table 6**. When re-examined in this way, differences in the distribution of Rif^R^ mutants in FL vs. GC samples were still apparent. For example, the Q137R and L489S mutations were each found in 2 separate FL PDFUs, but in zero GC PDFUs (**Table 6**). In contrast, mutations leading to Q469R predominated in both FL (6/6) and GC (5/6) PDFUs, suggesting that occurrence of this specific mutation is not responsive to spaceflight exposure (**Table 6**).

### Transitions vs. Transversion in FL and GC Samples

**FIGURE 4:**
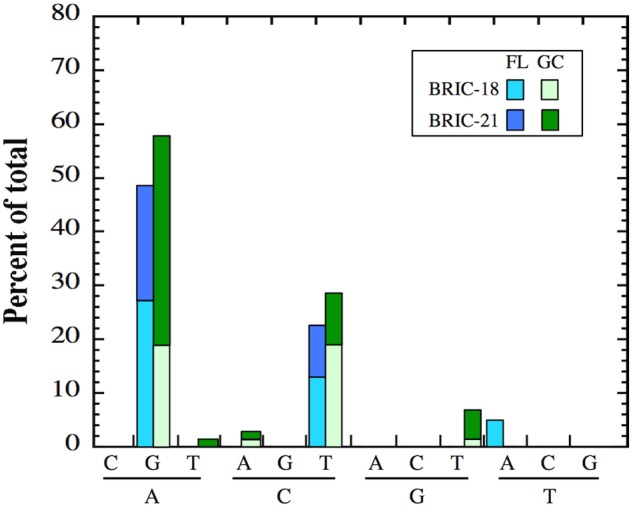
Frequency of transition and transversion mutations in FL (cyan and blue bars) and GC (light green and dark green bars) samples of *B. subtilis rpoB* in the BRIC-18 and BRIC-21 missions.

As reported above, differences were observed in the location of *rpoB* mutations in FL vs. GC samples in *B. subtilis* Rif^R^ mutants. These observations prompted us to examine the nature of nucleotide changes (i.e., transition vs. transversion mutations) in FL vs. GC samples from the BRIC-18 and BRIC-21 missions. In both FL and GC samples, A:G and C:T transitions predominated in both missions, and at relatively similar frequencies (**Figure 4**). In addition, GC samples exhibited A:T, C:A, and G:T transversions that were not found in FL samples; conversely, FL samples exhibited T:A transversions that were not found in GC samples (**Figure 4**). Thus, the spectrum of mutational classes (transitions vs. transversions) was also altered in FL vs. GC samples in both the BRIC-18 and BRIC-21 missions.

### Mapping Rif^R^ Mutations on the Structure of RpoB

**FIGURE 5:**
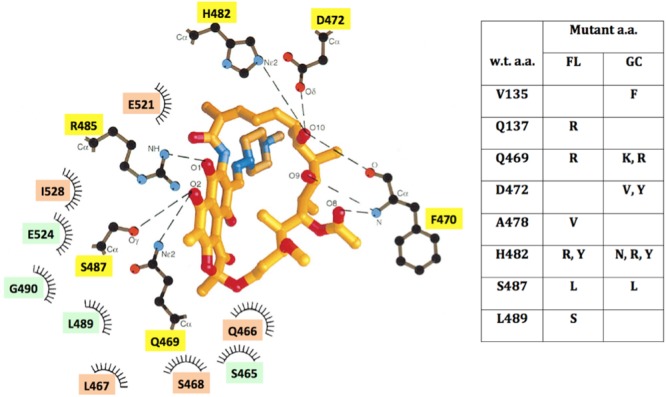
**(Left)** Schematic depiction of the Rif-binding pocket in the β-subunit of RNA polymerase, adapted from ref. (Campbell et al., 2001), using *B. subtilis* amino acid coordinates. Amino acids located within 6 Å of Rif are denoted which: make direct H-bonding contacts with Rif (yellow boxes); make hydrophobic contacts (orange boxes); or form part of the binding pocket (green boxes). **(Right)** Table of locations and identities of amino acid (a.a.) substitutions leading to Rif^R^ in FL and GC samples. See text for details.

Three-dimensional structures of RpoB elucidated from a number of bacteria (reviewed recently in Murakami, 2015) and supported by the crystal structure of RpoB in complex with Rif (Campbell et al., 2001), have revealed a great deal of structural conservation in the Rif-binding pocket. Rif binds in the concave surface of a roughly bowl-shaped depression in the RNA exit channel of RpoB, held in place both by direct H-bonding contacts and hydrophobic and ionic interactions with amino acids lining the pocket (**Figure 5**). Amino acids Q469, F470, D472, H482, R485, and S487 make direct H-bonds with Rif (Campbell et al., 2001), and are most frequently associated with Rif^R^ (Severinov et al., 1993; Campbell et al., 2001); indeed we observed that mutations at these positions led to Rif^R^ in FL and GC samples. Amino acids in the N-cluster do approach the Rif-binding pocket, but not within the 6 Å distance depicted in **Figure 5**. It is thought that substitution of amino acids containing relatively small side groups (V135, Q137) with bulkier side groups (F135, R137) can exert longer-range effects by distorting the binding pocket out of its optimum shape (Severinov et al., 1994).

## Discussion

In this communication we describe the results of two spaceflight experiments using *B. subtilis* in which growth, the frequency of mutation to Rif^R^, and the corresponding spectrum of mutations within the Rif^R^ regions of the *rpoB* gene, were measured.

### Growth in FL vs GC Samples

A number of experiments have been conducted in which various bacterial species have been cultivated under otherwise-matching conditions of hardware, media, inoculation, etc., and their growth, as measured by final cell density, has been compared in spaceflight vs. ground control samples. Some of these prior studies have shown increased final cell density during spaceflight, while others have found either no significant differences between spaceflight and 1 × *g* conditions (reviewed in Benoit and Klaus, 2007; Horneck et al., 2010; Kim et al., 2013; Rosenzweig et al., 2014; Coil et al., 2016), or even decreased growth in spaceflight (Fajardo-Cavazos and Nicholson, 2016). In both the BRIC-18 and BRIC-21 experiments described here, we measured no statistically significant difference in the final viable counts of *Bacillus subtilis* cells in FL vs. GC samples.

As in most previous spaceflight experiments, the design of BRIC canisters does not allow growth measurements to be taken during the experiment, thus it was not possible to determine growth rates. However, the growth rate of *B. subtilis* cells in spaceflight vs. matched ground control samples was directly measured in the SESLO (Space Environment Survivability of Living Organisms) payload of the O/OREOS nanosatellite, and in that experiment cells were observed to grow at a slower rate in spaceflight (Nicholson et al., 2011).

### Mutation Frequencies in FL vs. GC Samples

Although the human spaceflight environment in Low Earth Orbit is characterized by an increased flux of high-energy particles, and enhanced space radiation effects have been documented in astronauts and higher organisms (reviewed in De Micco et al., 2011; Maalouf et al., 2011), very few studies to date have tested the notion that exposure to the human spaceflight environment could lead to increased mutagenesis in microbes. In two prior studies, the mutation frequency was either found not to be different in FL vs. GC (Yatagai et al., 2000) or to be elevated ∼24-fold in FL samples (Fajardo-Cavazos and Nicholson, 2016). The results described here, from 2 separate spaceflight missions to the ISS (BRIC-18 and BRIC-21), showed that the frequency of mutation to Rif^R^ in *B. subtilis* was not significantly different in FL vs GC samples.

### Spectrum of *rpoB* Mutations in FL vs. GC

The BRIC-18 and BRIC-21 experiments reported here showed that the spectrum of mutations in *B. subtilis rpoB* leading to Rif^R^ was substantially different in FL vs. GC samples. Especially noteworthy were the mutations at Q137R and L489S which were identified only in FL samples; these alleles have never before been observed in *B. subtilis rpoB.* The results are in accord with the notion that the set of stresses defining an environment can elicit specific spectra of mutations. The observations from spaceflight reported here bolster the findings from prior ground-based experiments which showed that growth of *B. subtilis* under sporulating vs. non-sporulating conditions (Nicholson and Maughan, 2002) or aerobic vs. anaerobic conditions (Nicholson and Park, 2015), resulted in distinct spectra of mutations to Rif^R^. This phenomenon is not peculiar to *B. subtilis*; it has also been reported in *Staphylococcus aureus* (Didier et al., 2011), *Escherichia coli* (Lindsey et al., 2013; Maharjan and Ferenci, 2017b), *Pseudomonas aeruginosa* and *P. putida* (Jatsenko et al., 2010), and *Mycobacterium tuberculosis* (Jenkins et al., 2009) (reviewed in refs. Koch et al., 2014; Alifano et al., 2015). It is significant to note that several of the above bacteria have also been found in human spaceflight habitats (Venkateswaran et al., 2014; Checinska et al., 2015).

### Broader Implications

Because RNA polymerase is a single enzyme responsible for transcribing all genes in a bacterium, mutations in its subunits can have far-reaching consequences for global regulation of the microbial transcriptome. In particular, mutations in *rpoB* have been shown to cause Rif-independent physiological effects in a wide range of bacterial species (reviewed in ref. Koch et al., 2014). Such effects include: activation of cryptic metabolic capabilities (Perkins and Nicholson, 2008); alterations in the susceptibility to other classes of antibiotics (Kane et al., 1979; Kristich and Little, 2012); or production of various secondary metabolites (Hu et al., 2002). Furthermore, it has been shown that certain mutant alleles of *rpoB* actually enhance the fitness of the resulting Rif^R^ mutants under specific environmental stresses, such as growth under nutrient-limiting conditions (Maharjan and Ferenci, 2017a). These physiologic effects could have profound consequences for long-duration missions in space, as microbes will continue to evolve in human space habitats. We are currently pursuing the idea that mutations induced by the stress of spaceflight may provide enhanced evolutionary fitness in the spaceflight environment itself. Understanding microbial evolution in space is crucial to better cope with wide-ranging challenges, from biofilm formation to biocorrosion of materials to alterations in the human microbiome.

## Author Contributions

PF-C contributed in design of the investigation, execution of the experiments, and writing of the manuscript. JL contributed in execution of experiments and writing of the manuscript. WN contributed to the design of the investigation, design and execution of the experiments, interpretation of the data, and writing of the manuscript.

## Conflict of Interest Statement

The authors declare that the research was conducted in the absence of any commercial or financial relationships that could be construed as a potential conflict of interest.

## Footnotes

1 https://www.nasa.gov/content/journey-to-mars-overview

2 http://www.ebi.ac.uk/Tools/msa/clustalo/
